# Exosome-derived miR-548ag drives hepatic lipid accumulation via upregulating FASN through inhibition of DNMT3B

**DOI:** 10.1016/j.jlr.2025.100818

**Published:** 2025-05-06

**Authors:** Xiaolong Chu, Yanting Hou, Chaoling Peng, Wei Li, Maodi Liang, Jin Mei, Meiyu Qian, Juan Wang, Shibo Xu, Yidan Jiang, Xin Wen, Yao Chen, Fangyuan Yuan, Jianxin Xie, Cuizhe Wang, Jun Zhang

**Affiliations:** 1 Department of Basic Medicine, Medical College, Shihezi University, Shihezi, Xinjiang, China; 2 Department of Medical Genetics, Medical College of Tarim University, Alar, Xinjiang, China; 3 Laboratory of Xinjiang Endemic and Ethic Diseases, Shihezi University, Shihezi, Xinjiang, China; 4 First Affiliated Hospital of Shihezi University School of Medicine, Shihezi, Xinjiang, China

**Keywords:** obesity, miR-548ag, DNMT3B, FASN, MASLD

## Abstract

Metabolic dysfunction-associated steatotic liver disease (MASLD) is the leading cause of chronic liver disease worldwide. This study investigates the role of serum miR-548ag in regulating lipid metabolism and its contribution to MASLD in obesity. We found that miR-548ag levels were significantly elevated in the serum of both obese and MASLD patients and positively correlated with body mass index, fasting plasma glucose, triglycerides, total cholesterol, LDL, HDL, aspartate aminotransferase, and alanine aminotransferase levels. Additionally, miR-548ag expression was significantly higher in the liver and abdominal adipose tissue of obese individuals than those of normal weight. In vitro studies in HepG2 and L02 cells, along with previous findings, demonstrated that miR-548ag promotes fatty acid synthase (FASN) expression by inhibiting DNA methyltransferase 3B (DNMT3B), thereby enhancing lipid synthesis. This was confirmed in two mouse models: one with tail vein injections of miR-548ag mimic/inhibitor adeno-associated viruses and another with tail vein injections of exosomes from serum of normal-weight and obese individuals. Both models showed that miR-548ag upregulated FASN through DNMT3B inhibition, leading to increased lipid synthesis and larger hepatic lipid droplets, effects that were reversed by miR-548ag inhibition. Together, this study revealed a significant increase in the levels of exosome miR-548ag in the serum of obese patients, which reaches the liver through blood circulation. In the liver, miR-548ag can target and inhibit DNMT3B, upregulate FASN expression, and increase hepatic lipid synthesis, thereby promoting the development of MASLD.

## Graphical Abstract

**Figure:**
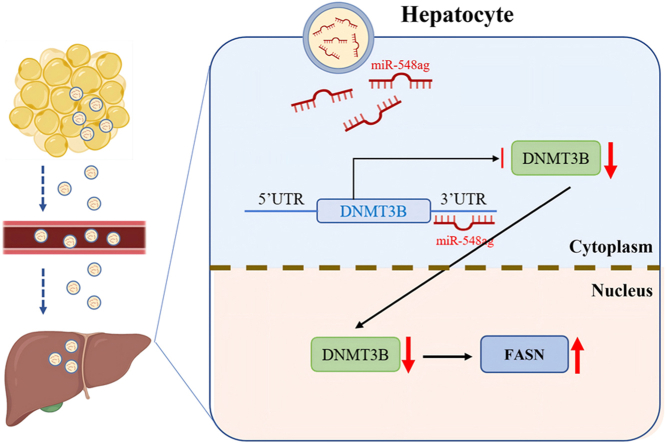

Metabolic dysfunction-associated steatotic liver disease (MASLD) is the most common chronic liver disease globally (1), affecting over a quarter of the world’s adult population. Obesity is a major risk factor for MASLD, with prevalence rates as high as 50%–70% in obese individuals. In China, more than 310 million people are estimated to be affected by MASLD (2, 3, 4, 5, 6). Approximately 5%–20% of MASLD cases progress to metabolic dysfunction-associated steatohepatitis (1, 7), a severe form characterized by hepatic inflammation, ballooning degeneration, and fibrosis, which can eventually lead to cirrhosis or hepatocellular carcinoma (8, 9). Despite significant research efforts, the precise molecular mechanisms underlying obesity-induced MASLD remain incompletely understood.

Adipose tissue, traditionally viewed as an energy reservoir, is now recognized as a critical endocrine organ. It secretes numerous hormones and cytokines, collectively termed adipokines, which regulate energy intake, storage, and expenditure, thus influencing whole-body metabolism (10, 11, 12). MicroRNAs (miRNAs) have emerged as novel adipokines that not only modulate gene expression within cells but also circulate in exosomes, influencing gene regulation in distant target tissues (13, 14, 15, 16, 17, 18). In obesity, dysregulated miRNA expression can induce insulin resistance and type 2 diabetes by altering glucose metabolism in key metabolic tissues such as adipose, liver, and muscle (19, 20, 21, 22, 23, 24, 25, 26, 27). Circulating miRNAs primarily derive from adipose tissue exosomes. Our previous work demonstrated that elevated palmitic acid levels in obesity trigger exosomal miRNA release from adipocytes via NF-κB and endoplasmic reticulum stress pathways (28). Studies have shown that miR-99b from brown adipose tissue exosomes reduces hepatic FGF21 expression, improving glucose tolerance in mice (29), while miR-222 from gonadal white adipose tissue exosomes suppresses IRS1 and AKT phosphorylation, thereby impairing insulin sensitivity and glucose tolerance (30). Furthermore, exosomal miR-122, derived from adipocytes, promotes MASLD progression by targeting Sirt1 (31). Research on the role of miRNAs in lipids metabolism has increased, resulting in an expansion of the application prospects of miRNAs as diagnostic markers and therapeutic targets in the treatment of obesity-related metabolic diseases.

Our previous work demonstrated that serum levels of adipose-derived miR-548ag correlate positively with blood glucose and lipid profiles and that miR-548ag induces insulin resistance by targeting and inhibiting hepatic DNA methyltransferase 3B (DNMT3B), leading to increased DPP4 expression (32). However, the role of miR-548ag in lipid metabolism remains unexplored. Fatty acid synthase (FASN), a key enzyme in de novo lipogenesis, is a critical regulator of hepatic lipid accumulation and has been identified as a potential therapeutic target for MASLD (33). In this study, we found that serum miR-548ag levels were significantly elevated in MASLD patients compared to normal-weight individuals and correlated with BMI, fasting blood glucose, lipid profiles, and liver enzymes (ALT and AST). Bioinformatics analysis predicted DNMT3B and FASN as downstream targets of miR-548ag. We further demonstrate that miR-548ag expression and release from adipose-derived exosomes are increased in obesity and, through inhibition of DNMT3B, upregulate FASN expression, resulting in enhanced hepatic lipid accumulation. Importantly, miR-548ag inhibition significantly reduces body weight and hepatic lipid levels in obese mice.

## Materials and Methods

### Serum and tissue sample collection

Serum samples were collected from 83 individuals in Xinjiang Province, China. General demographic and clinical data, including age, height, weight, waist circumference, BMI, fasting plasma glucose (FPG), and serum triglyceride (TG), total cholesterol (TC), high-density lipoprotein cholesterol, and low-density lipoprotein cholesterol levels, were recorded. Participants were categorized into three groups: the normal-weight group (NC group, n = 41, 18.5 kg/m^2^ ≤ BMI ≤ 24 kg/m^2^), the obese group (OB group, n = 18, BMI ≥ 28 kg/m^2^), and the MASLD group (n = 24), clinically diagnosed with MASLD.

Abdominal adipose tissue samples were obtained from 12 individuals, including 6 normal-weight and 6 overweight/obese participants, at the First Affiliated Hospital of Shihezi University School of Medicine. Similarly, liver tissue samples were collected from 12 individuals, comprising 6 normal-weight and 6 overweight/obese participants.

### MASLD diagnosis and participant inclusion criteria

The diagnosis of MASLD was based on imaging findings combined with biochemical markers, following these inclusion criteria: male-to-female ratio of 1:1; age range of 18–70 years; no history of medication use in the past month; no recent excessive alcohol consumption or binge eating in the two weeks prior to the study.

Exclusion criteria included the following: history of chronic alcohol consumption exceeding 5 years (≥40 g/day ethanol for men or ≥20 g/day for women) or acute heavy drinking within the past 2 weeks (>80 g/day ethanol); viral hepatitis; autoimmune liver diseases or genetic disorders; history of drug-induced liver injury; history of total parenteral nutrition; history of diabetes mellitus or hyperthyroidism; use of medications affecting insulin secretion or sensitivity; presence of malignant tumors or other progressive terminal illnesses.

### Ethical approval

All human-related studies in this research strictly adhere to the ethical principles of the Declaration of Helsinki. All participants provided informed consent before enrollment, acknowledging that their clinical information and biological samples would be used for research and publication purposes. The study protocol and consent procedures were approved by the Medical Ethics Committee of the First Affiliated Hospital of Shihezi University School of Medicine (Approval Number: KJ2023-083-01). The weights, waist measurements, glycemic index values, and BMIs of all included individuals were obtained. The levels of FPG, TC, TG, HDL, and LDL were detected using an automated biochemistry analyzer (Mindray).

### Animal care

Male C57BL/6 mice were purchased from the SLAC Laboratory Animal Co., Ltd. (Hunan, China). The mice were housed in a controlled environment with a temperature of 22–24°C, humidity of 40%–50%, and a 12-h light/dark cycle. All animal care and handling procedures were carried out in accordance with international laws and regulations. The animal experiments in this study were approved by the Animal Ethics Committee of the First Affiliated Hospital of Shihezi University (Approval No. A2019-087-01).

### Exosome isolation and characterization

Human serum or cell culture supernatants were transferred to a new centrifuge tube and centrifuged at 4°C, 2000g for 10 min. The supernatant was carefully transferred to a new tube and centrifuged again at 4°C, 10,000*g* for 30 min to remove larger vesicles. The resulting supernatant was then transferred to a new tube and subjected to ultracentrifugation at 4°C, 110,000*g* for 70 min using an ultracentrifuge rotor. The supernatant was discarded, and the exosome pellet was resuspended in prechilled PBS. The exosomes were centrifuged again at 4°C, 110,000*g* for 70 min, and the supernatant was removed. The pellet was resuspended in an appropriate amount of PBS. Exosomes were identified and characterized by transmission electron microscopy, nanoparticle tracking analysis, and Western blot analysis of exosomal markers. The isolated exosomes should be used immediately for downstream experiments or stored at −80°C.

### AAV8-mediated tail vein injection in mice

Eighteen 4-week-old male C57BL/6 mice were allowed to acclimate for 1 week, followed by an 8-weeks high-fat diet (HFD) to induce obesity. These mice were then randomly assigned to one of three groups: the control group (tail-vein injection of AAV8-*miR-548ag* control, 1 × 10^12^ vg/ml, n = 6), the miR-548ag overexpression group (tail-vein injection of AAV8-*miR-548ag* mimic, 1 × 10^12^ vg/ml, n = 6), and the miR-548ag inhibition followed by overexpression group (tail-vein injection of AAV8-*miR-548ag* inhibitor at week 7, followed by tail-vein injection of AAV8-*miR-548ag* mimic at week 9, 1 × 10^12^ vg/ml, n = 6). The mice were maintained on a HFD until week 14. The AAV8 construct has the following structure: ITR-*U6*-*miR-548ag* mimic/inhibitor-*CMV*-*mCherry*-*hGHpa*-ITR. This construct was designed to express miR-548ag mimic or inhibitor under the control of the *U6* promoter, with *mCherry* as a reporter gene driven by the *CMV* promoter for visualization of transduction efficiency. The construct was commercially synthesized and packaged into AAV8 capsids by GenePharma Co., Ltd. (Shanghai, China).

### Mouse tail vein injection of human serum exosomes

Eighteen 4-week-old male C57BL/6 mice were acclimatized for one week before being fed a HFD for 8 weeks to establish a diet-induced obesity model. Following this, the mice were randomly divided into three groups:

(1) Normal weight (NW) exo + AAV8-*miR-548ag* inhibitor control group (n = 6): In the seventh week, these mice received tail vein injections of AAV8-*miR-548ag* inhibitor control, followed by tail vein injections of normal-weight human serum exosomes (30 μg per injection, twice a week) starting from week 9. The injections were continued until week 14.

(2) OB exo + AAV8-*miR-548ag* inhibitor control group (n = 6): In this group, mice were first administered AAV8-*miR-548ag* inhibitor control via tail vein injection in week 7, followed by injections of exosomes derived from obese human serum (30 μg per injection, twice a week) beginning in week 9, continuing until week 14.

(3) OB exo + AAV8-*miR-548ag* inhibitor group (n = 6): Similar to the second group, these mice received tail vein injections of AAV8-*miR-548ag* inhibitor in week 7. From week 9 onwards, they were treated with exosomes derived from obese human serum (30 μg per injection, twice a week), continuing until week 14.

The dose of 30 μg exosomes was determined based on previous studies demonstrating the efficacy and safety of this dose in mouse models for metabolic studies. Specifically, the quantity of circulating extracellular vesicles in obese mice was found to be approximately 30 μg per mouse, which served as a physiological reference for our dosing strategy (34). Specifically, 30 μg refers to the total protein content of the exosomes, which was quantified using the BCA protein assay.

### IVIS imaging

After 2 weeks of tail-vein injection with mCherry-labeled AAV8-*miR-548ag* mimic/inhibitor, in vivo imaging was performed using the IVIS® Lumina II system (PerkinElmer, Thermo Fisher Scientific) to assess the distribution and localization of the AAV8 vector in mice. For exosome tracking, exosomes were labeled with DiD (Beyotime, C1995S) and injected via tail vein into mice. Twelve hours postinjection, the distribution of exosomes throughout the body and in specific organs was evaluated using the IVIS® Lumina II system.

### Oil Red O staining of mouse liver tissue

Oil Red O staining was performed to assess lipid accumulation in mouse liver tissue. The Oil Red O solution (purchased from Solarbio Technology Co., Ltd., Beijing, China) was prepared as a 60% working solution, heated in a 60–70°C water bath for 30 min, and then filtered through filter paper after natural cooling. Additionally, 60% isopropanol was prepared for the differentiation step. Frozen liver tissue sections were retrieved from −20°C and allowed to warm to room temperature for 5–10 min. Once at room temperature, the sections were gently immersed in the Oil Red O working solution for 8–10 min (in the dark). Following staining, sections were briefly immersed in two containers of 60% isopropanol for 3–5 s to differentiate the staining. The sections were then washed in two changes of pure water for 10 s each. Nuclei were stained with hematoxylin, followed by water wash, bluing, and a final wash. After partial drying, the sections were mounted using glycerol-gelatin mounting medium.

### H&E staining of mouse liver tissue

Paraffin-embedded liver tissue sections were deparaffinized and rehydrated by sequential immersion in xylene I (10 min), xylene II (10 min), anhydrous ethanol I (5 min), anhydrous ethanol II (5 min), 95% ethanol (5 min), 90% ethanol (5 min), 80% ethanol (5 min), 70% ethanol (5 min), and distilled water. Sections were then stained with Harris hematoxylin for 3–8 min, followed by rinsing with tap water. Differentiation was performed using 1% hydrochloric acid in ethanol for a few seconds, followed by a rinse with tap water and bluing in 0.6% ammonia water. Sections were washed under running water. The sections were then stained with eosin for 1–3 min. After dehydration, the sections were sequentially immersed in 95% ethanol I (5 min), 95% ethanol II (5 min), anhydrous ethanol I (5 min), anhydrous ethanol II (5 min), xylene I (5 min), and xylene II (5 min) for dehydration and transparency. After briefly air-drying, sections were mounted with neutral resin.

### Measurement of TG content in mouse liver tissue

The liver tissue was weighed and homogenized in a 1:9 ratio of tissue (g) to anhydrous ethanol (mL) using a mechanical homogenizer in an ice-water bath. The homogenate was centrifuged at 2,500 rpm for 10 min, and the supernatant was collected for TG content measurement. TG levels were quantified using a TG assay kit (Nanjing Jiancheng Bioengineering Institute, China), following the manufacturer's protocol.

### Glucose tolerance test in mice

After a 12-h fasting period, the body weight of the mice was recorded, and the glucose solution for intraperitoneal injection was prepared at a dose of 2 g/kg body weight. A small portion (1–2 mm) of the tail tip was clipped to collect blood for glucose measurements. Blood glucose levels were measured at 0, 5, 15, 30, 60, 90, and 120 min after glucose injection. The area under the glucose curve for each mouse was calculated using GraphPad Prism software.

### Insulin tolerance test in mice

After a 4-h fasting period, the body weight of the mice was recorded. A small portion (1–2 mm) of the tail tip was clipped, and fasting blood glucose levels were measured using a glucometer. This value was considered as the 0-min baseline glucose level. Following a 30-min adaptation period, the mice were administered intraperitoneal glucose injections (0.01 ml per gram body weight). Blood glucose levels were measured at 15, 30, 60, and 120 min postinjection. The area under the glucose curve was calculated for each mouse using GraphPad Prism software.

### Cell culture

Human hepatocellular carcinoma HepG2 and human normal liver L02 cells were obtained from the Cell Bank of the Chinese Academy of Sciences (Shanghai, China). Cells were cultured in DMEM medium (containing 4.5 g/L D-glucose), supplemented with 10% FBS and 1% penicillin-streptomycin mixture (100X), and incubated at 37°C in a humidified incubator with 5% CO2. After transfection, cells were harvested for RNA and protein extraction or subjected to Oil Red O staining and TG level measurement.

### Cell transfection

For transfection of miR-548ag mimic, miR-548ag inhibitor, *DNMT3B* siRNA, or *DNMT3B* overexpression plasmids, Lipofectamine 2000 was used for the former two, and Lipofectamine 3,000 was used for the latter. HepG2 cells were transfected with miR-548ag mimic (50 nM), miR-548ag inhibitor (100 nM), *DNMT3B* siRNA (80 nM), or *DNMT3B* overexpression plasmid (4 μg/ml). L02 cells were transfected with miR-548ag mimic (50 nM), miR-548ag inhibitor (100 nM), *DNMT3B* siRNA (40 nM), or *DNMT3B* overexpression plasmid (1 μg/ml). After 4–6 h of transfection, the medium was replaced with DMEM containing 10% FBS, and cells were incubated for an additional 24 h before RNA and protein extraction or other downstream analyses. The miR-548ag mimic, miR-548ag inhibitor, and *DNMT3B* siRNA used in this study were synthesized by Shanghai GenePharma Co., Ltd., and the *DNMT3B* overexpression plasmid was obtained from Beijing HeSheng Biotechnology Co., Ltd.

### Treatment of cells with FFA solution

Stock solutions of palmitic acid (PA) and oleic acid (OA) were prepared separately at 40 mM concentration in 0.1 M NaOH and complexed with 10% fatty acid-free bovine serum albumin at 37°C for 4 h to ensure proper solubilization. Immediately before treatment, PA and OA stock solutions were mixed at a 1:2 ratio (PA:OA) to prepare the FFA mixture. For cell treatment, this mixture was diluted in culture medium to achieve a final concentration of 1.5 mmol/L total FFAs (containing 0.5 mmol/L PA and 1.0 mmol/L OA). Cells were then incubated with the FFA-containing medium for 24 h to mimic lipid overload conditions.

### Oil Red O staining of cells

Following cell fixation with a fixative solution, Oil Red O staining solution was added, and the cells were incubated at 37°C in a water bath for staining. After staining, the cells were washed, and lipid droplets were observed under a microscope at 400× magnification. Images were captured for documentation. For quantitative analysis, the Oil Red O dye attached to the lipid droplets was eluted with isopropanol at 37°C in a water bath. The eluted solution was measured for absorbance at 570 nm using a microplate reader.

### Detection of TG content in cells

To prepare the Triton X-100 lysis buffer, 750 μl of Triton X-100 was added to 50 ml of distilled water. After the treatment, the cells were washed twice with 1 ml of 1×PBS to remove residual media. Then, 200–500 μl of the prepared Triton X-100 lysis buffer was added to each well, and the cells were lysed at 4°C for 1 h. Afterward, the lysed cell fragments were carefully pipetted and transferred to EP tubes. The TG content in the lysates was measured using the TG assay kit from Nanjing Jianchen Biological Engineering Institute.

### Western blotting and antibodies

Cell lysis was performed using RIPA buffer supplemented with PMSF (1:100 ratio) to extract total cellular proteins. Equal amounts of protein were loaded for electrophoresis and subjected to immunoblotting with antibodies against GAPDH, DNMT3B, and FASN. The GAPDH antibody was purchased from Beijing Zhongshan Jinqiao Corporation (China), while DNMT3B and FASN antibodies were sourced from Abcam.

### RNA extraction and quantitative real-time PCR

miRNA extraction and quantification: For human and mouse serum samples, miRNAs were isolated using the miRcute Serum/Plasma miRNA Isolation Kit (TIANGEN, Beijing, China), with 1 pmol of Cel-miR-39 spiked into the lysis buffer as an external reference. For human and mouse liver tissues and cell lines, total RNA (including miRNAs) was extracted using TRIzol reagent (Invitrogen). Exosome-derived miRNAs were similarly extracted using TRIzol reagent, with 1 pmol of Cel-miR-39 added as an external reference during lysis. Reverse transcription was performed using the miRcute Plus miRNA First-Strand cDNA Kit (TIANGEN), followed by qRT-PCR using the miRcute Plus miRNA qPCR Kit (TIANGEN). Cel-miR-39 served as the external reference for serum and exosome samples, while U6 snRNA was used as the internal reference for cells and tissues.

mRNA extraction and quantification: Total RNA was extracted from all samples using TRIzol reagent (Invitrogen). RNA concentration and purity were determined by spectrophotometric analysis (NanoDrop 2000, Thermo Fisher Scientific). For complementary DNA synthesis of DNMT3B, FASN and GAPDH, 1 μg of total RNA was reverse transcribed using the cDNA Reverse Transcription Kit (Thermo Fisher Scientific) following the manufacturer's protocol. Quantitative real-time PCR was performed using the QuantiTect SYBR Green PCR Kit (QIAGEN, Germany) on a QIAGEN real-time PCR system. All primer sequences are provided in Supplementary Table S1.

### Statistical analysis

Statistical analysis was performed using SPSS 17.0 software. If the data were normally distributed, a *t* test was used for comparisons between groups. For non-normally distributed data, nonparametric rank sum tests were performed. A *P*-value of <0.05 was considered statistically significant.

## Results

### miR-548ag exhibits marked upregulation in individuals with obesity and MASLD

**Fig. 1:**
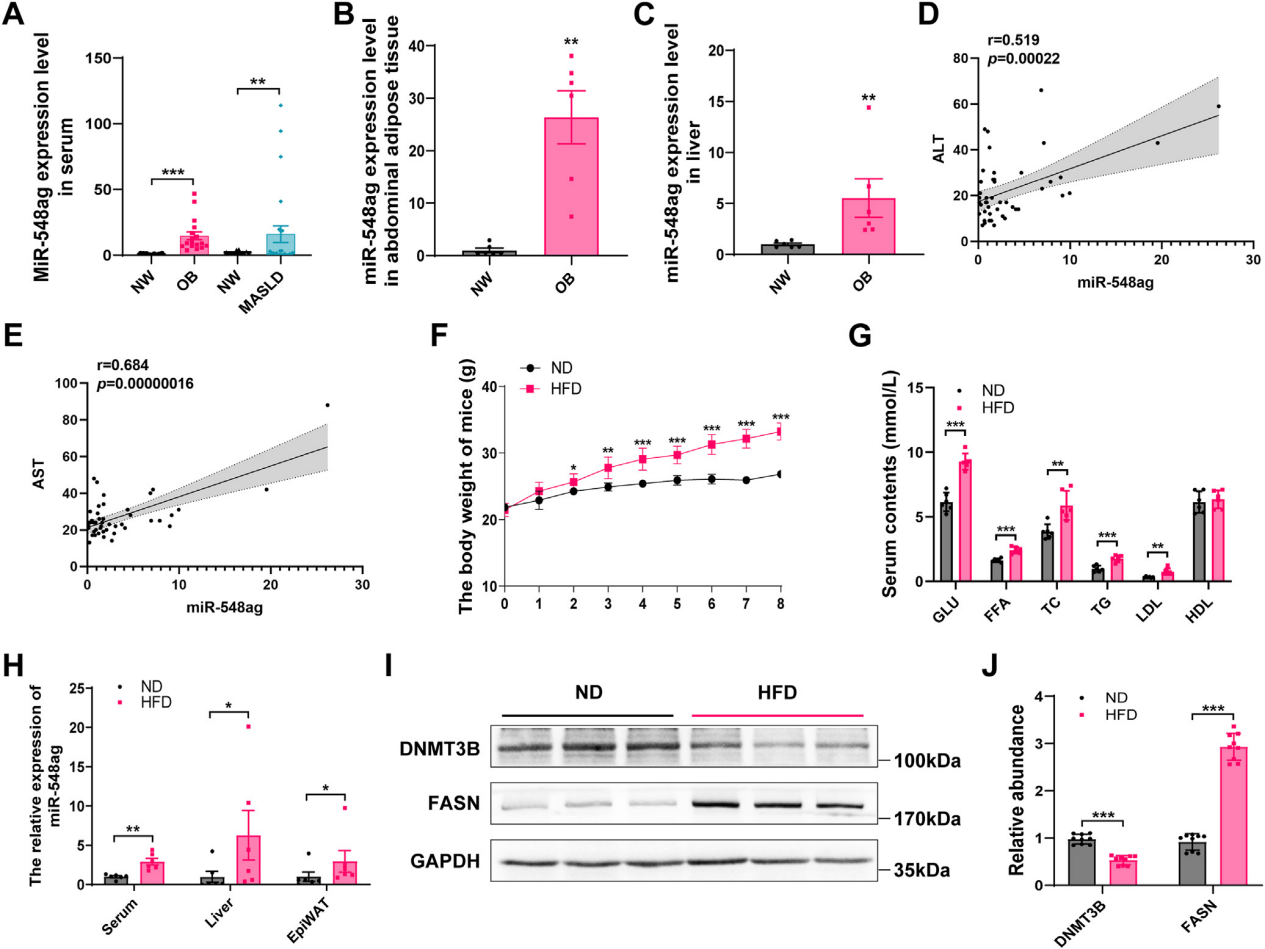
Effect of obesity on the expression level of miR-548ag. A: Serum levels of miR-548ag in obese and MASLD subject individuals (NW = 42, OB = 17, MASLD = 25). B, C: Expression of miR-548ag in abdominal adipose tissue and liver tissue of obese individuals (n = 6). D, E: Correlation analysis of serum miR-548ag level with ALT and AST (n = 46). F: Mouse weight (n = 6). G: Blood glucose and lipid levels in mice (n = 6). H: Serum, liver, and epididymal adipose tissue miR-548ag in mice (n = 6). I, J: Protein expression and quantification of DNMT3B and FASN in mouse liver (The *P*-values by the *t* test and nonparametric rank sum test are indicated. Data presented as mean ± sem. ∗*P* < 0.05, ∗∗*P* < 0.01, and ∗∗∗*P* < 0.001 indicate a significant difference).

The levels of miR-548ag in both serum, adipose tissues and liver tissues were significantly elevated in obese and MASLD subjects compared to individuals with normal body weight (Fig. 1A–C). Serum miR-548ag levels positively correlated with BMI, FPG, TG, TC, LDL, and HDL (Supplementary Table S2), as well as ALT and AST (Fig. 1D, E). TargetScan database analysis combined with KEGG pathway enrichment revealed that the metabolic functions of miR-548ag are primarily enriched in lipid metabolism pathways (Supplemental Fig. S1A), identifying *DNMT3B* and *FASN* as potential downstream target genes (Supplemental Fig. S1B–D).

In an HFD-induced obesity mouse model successfully established for this study (Fig. 1F–G), miR-548ag levels were significantly increased in the serum, liver, and epididymal adipose tissues of HFD mice compared to normal diet mice (Fig. 1H). Moreover, protein expression of DNMT3B in the liver tissues of HFD mice was significantly decreased, while FASN protein expression was markedly elevated (Fig. 1I, J).

Following the protocol described in a recent Nature article published in August 2024 (35), we fed C57BL/6 mice a HFD for 8 weeks. The mice were then divided into three groups: (1) a normal feeding group, (2) a 24-h fasting group, and (3) a 24-h fasting followed by 24-h refeeding group. After the respective treatments, the mice were sacrificed, and serum and liver tissues were collected. We subsequently measured the expression levels of miR-548ag and DNMT3B, and the results are as follows:

In the 24-h fasting group, we observed a significant downregulation of miR-548ag and a corresponding upregulation of DNMT3B in the liver compared to the normal feeding group. This suggests that fasting inhibits miR-548ag expression, which in turn promotes DNMT3B. Conversely, in the 24-h fasting followed by 24-h refeeding group, miR-548ag levels increased significantly, while DNMT3B expression was restored to levels comparable to those in the normal feeding group. These findings indicate that miR-548ag and DNMT3B are dynamically regulated in response to nutritional status, with fasting suppressing miR-548ag and enhancing DNMT3B expression, while refeeding reverses these effects (Supplemental Fig. S2A–D).

### miR-548ag regulates DNMT3B and FASN expression, influencing lipid accumulation in hepatocytes

**Fig. 2:**
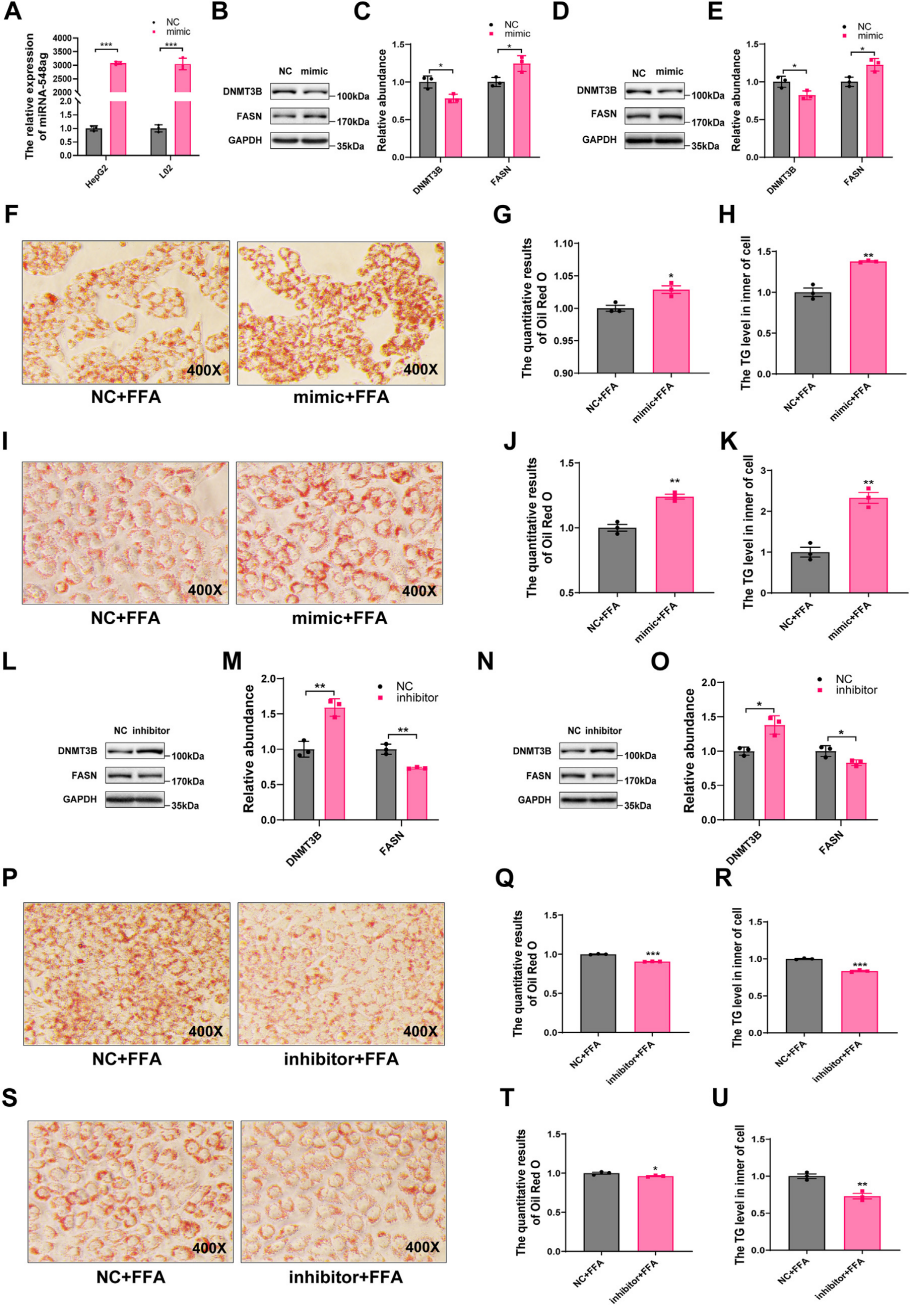
Effect of miR-548ag on the expression of DNMT3B and FASN. A: Upregulation of miR-548ag expression levels in HepG2 and L02 cells using 50 nM miR548ag mimic, respectively. B, C: Protein expression levels of DNMT3B and FASN in HepG2 cells after overexpression of miR-548ag. D, E: Protein expression levels of DNMT3B and FASN in L02 cells after overexpression of miR-548ag. F, G: Oil Red O staining and quantitative analysis of HepG2 cells after cotreatment with FFA mixture and miR-548ag overexpression (400×). H: TG content in HepG2 cells after cotreatment with FFA mixture and miR-548ag overexpression. I, J: Oil Red O staining and quantitative analysis of L02 cells after cotreatment with FFA mixture and miR-548ag overexpression (400×). K: TG content in L02 cells after cotreatment with FFA mixture and miR-548ag overexpression. L, M: HepG2 cells were transfected with 100 nM miR-548ag inhibitor for 24 h, respectively, and the protein expression levels of DNMT3B and FASN were detected in the cells. N, O: L02 cells were transfected with 100 nM miR-548ag inhibitor for 24 h, respectively, and the protein expression levels of DNMT3B and FASN were detected in the cells. P, Q: Oil Red O staining and quantitative analysis of HepG2 cells after cotreatment with FFA mixture and miR-548ag inhibition(400×). R: TG content in HepG2 cells after cotreatment with FFA mixture and miR-548ag inhibition. S, T: Oil Red O staining and quantitative analysis of L02 cells after cotreatment with FFA mixture and miR-548ag inhibition (400×). U: TG content in L02 cells after cotreatment with FFA mixture and miR-548ag inhibition (Each experiment was repeated at least three times. The *P*-values by the *t* test and nonparametric rank sum test are indicated. Data presented as mean ± sem. ∗*P* < 0.05, ∗∗*P* < 0.01, and ∗∗∗*P* < 0.001 indicate a significant difference).

To elucidate the regulatory role of miR-548ag on DNMT3B and FASN, we transfected HepG2 and L02 hepatocytes with a miR-548ag mimic. Following transfection, the protein expression of DNMT3B was significantly reduced, while FASN expression was markedly elevated (Fig. 2A–E). Furthermore, in HepG2 and L02 cells treated with a 1.5 mmol/L FFA mixture (PA: OA = 1: 2), overexpression of miR-548ag resulted in a pronounced increase in intracellular lipid droplet accumulation and TG content (Fig. 2F–K).

Conversely, transfection of miR-548ag inhibitors into HepG2 and L02 cells produced opposing effects. Specifically, DNMT3B protein levels were significantly elevated, while FASN expression was markedly reduced (Fig. 2L–O). When miR-548ag inhibitors were combined with FFA mixture treatment, the lipid droplet accumulation and TG content in hepatocytes were significantly diminished compared to control groups (Fig. 2P–U).

These findings reveal that miR-548ag regulates DNMT3B and FASN protein expression, thereby driving lipid accumulation in response to lipogenic stimuli. This underscores the potential of miR-548ag as a promising molecular target for modulating lipid metabolism in hepatocytes.

### The effects of miR-548ag are mediated by DNMT3B and FASN

**Fig. 3:**
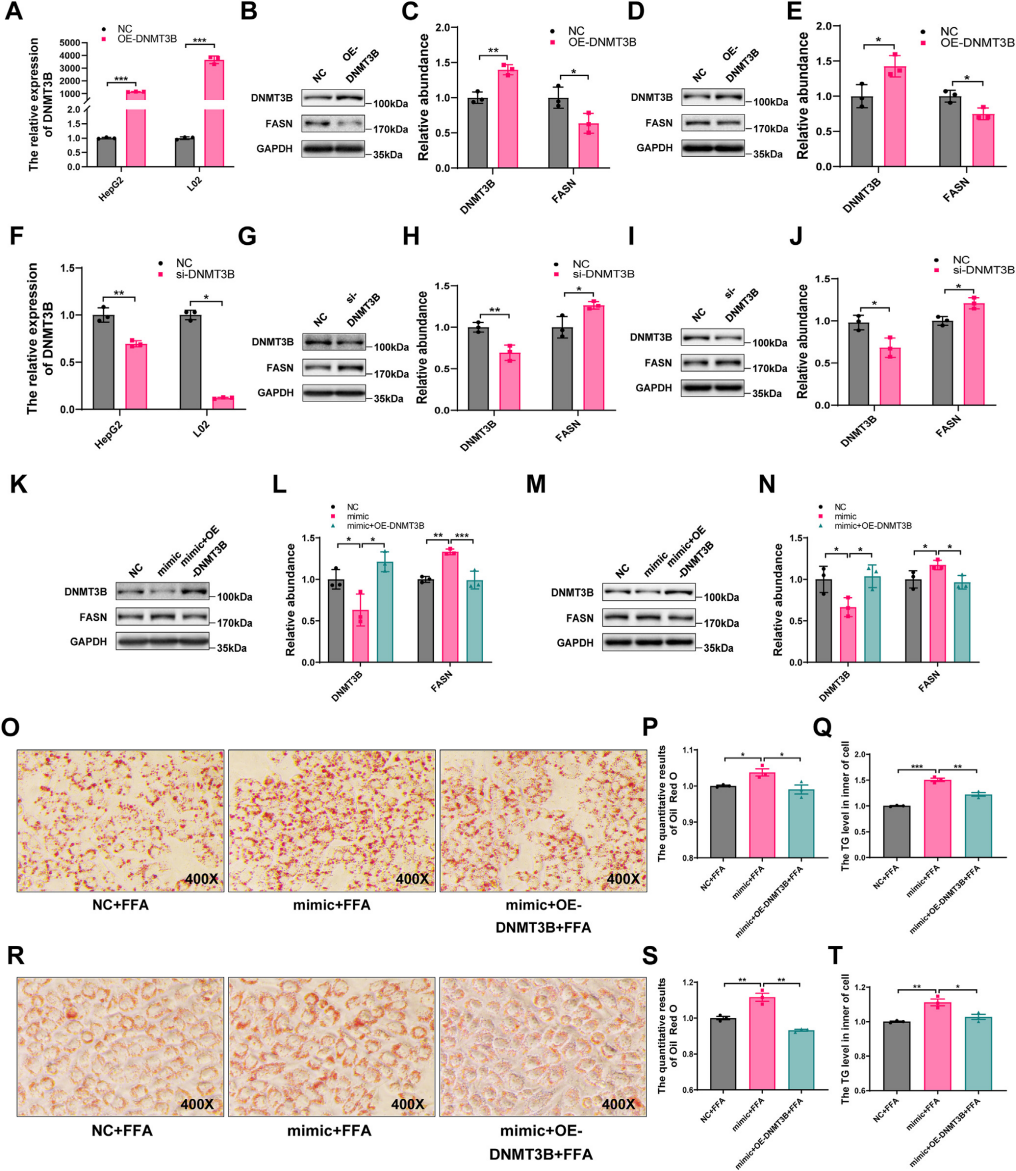
Rescue experiments of HepG2 and L02 cells. A: The expression levels of DNMT3B in HepG2 and L02 cells were upregulated using 4 μg/ml and 1 μg/ml DNMT3B overexpression plasmid, respectively. B, C: Protein expression levels of DNMT3B and FASN in HepG2 cells after DNMT3B overexpression. D, E: Protein expression levels of DNMT3B and FASN in L02 cells after DNMT3B overexpression. F: DNMT3B expression levels in HepG2 and L02 cells were downregulated using 80 nM and 40 nM DNMT3B interference fragments, respectively. G, H: Protein expression levels of DNMT3B and FASN in HepG2 cells after DNMT3B knockdown. I, J: Protein expression levels of DNMT3B and FASN in L02 cells after DNMT3B knockdown. K, L: Protein expression levels of DNMT3B and FASN in HepG2 cells after co-overexpression of miR-548ag and DNMT3B. M, N: Protein expression levels of DNMT3B and FASN in L02 cells after co-overexpression of miR-548ag and DNMT3B. O, P: Oil Red O staining and quantification results in HepG2 cells after cotreatment with FFA mixture combined with miR-548ag overexpression and DNMT3B upregulation (400×). Q: TG content in HepG2 cells after cotreatment with FFA mixture combined with miR-548ag overexpression and DNMT3B upregulation. R, S: Oil Red O staining and quantification results in L02 cells after cotreatment with FFA mixture combined with miR-548ag overexpression and DNMT3B upregulation (400×). T: TG content in L02 cells after cotreatment with FFA mixture combined with miR-548ag overexpression and DNMT3B upregulation (Each experiment was repeated at least three times. The *P*-values by the *t* test and nonparametric rank sum test are indicated. Data presented as mean ± sem. ∗*P* < 0.05, ∗∗*P* < 0.01, and ∗∗∗*P* < 0.001 indicate a significant difference).

To investigate the regulatory relationship between DNMT3B and FASN, we first overexpressed DNMT3B in hepatocytes. This led to a significant reduction in FASN protein levels in both HepG2 and L02 cells (Fig. 3A–E). Conversely, silencing DNMT3B using a targeted siRNA resulted in a marked increase in FASN protein expression (Fig. 3F–J).

Building on these observations, we examined the interplay between miR-548ag and DNMT3B. Overexpression of miR-548ag significantly suppressed DNMT3B protein levels while enhancing FASN protein expression compared to the negative control group. Notably, co-overexpression of DNMT3B reversed the miR-548ag–mediated upregulation of FASN in hepatocytes, restoring FASN levels to baseline (Fig. 3K–N).

Overexpression of miR-548ag led to a significant increase in intracellular lipid droplet accumulation and TG content. However, simultaneous upregulation of DNMT3B effectively counteracted these changes, reversing the miR-548ag–induced lipid synthesis in hepatocytes (Fig. 3O–T).

### miR-548ag promotes hepatic steatosis in HFD-fed mice

**Fig. 4:**
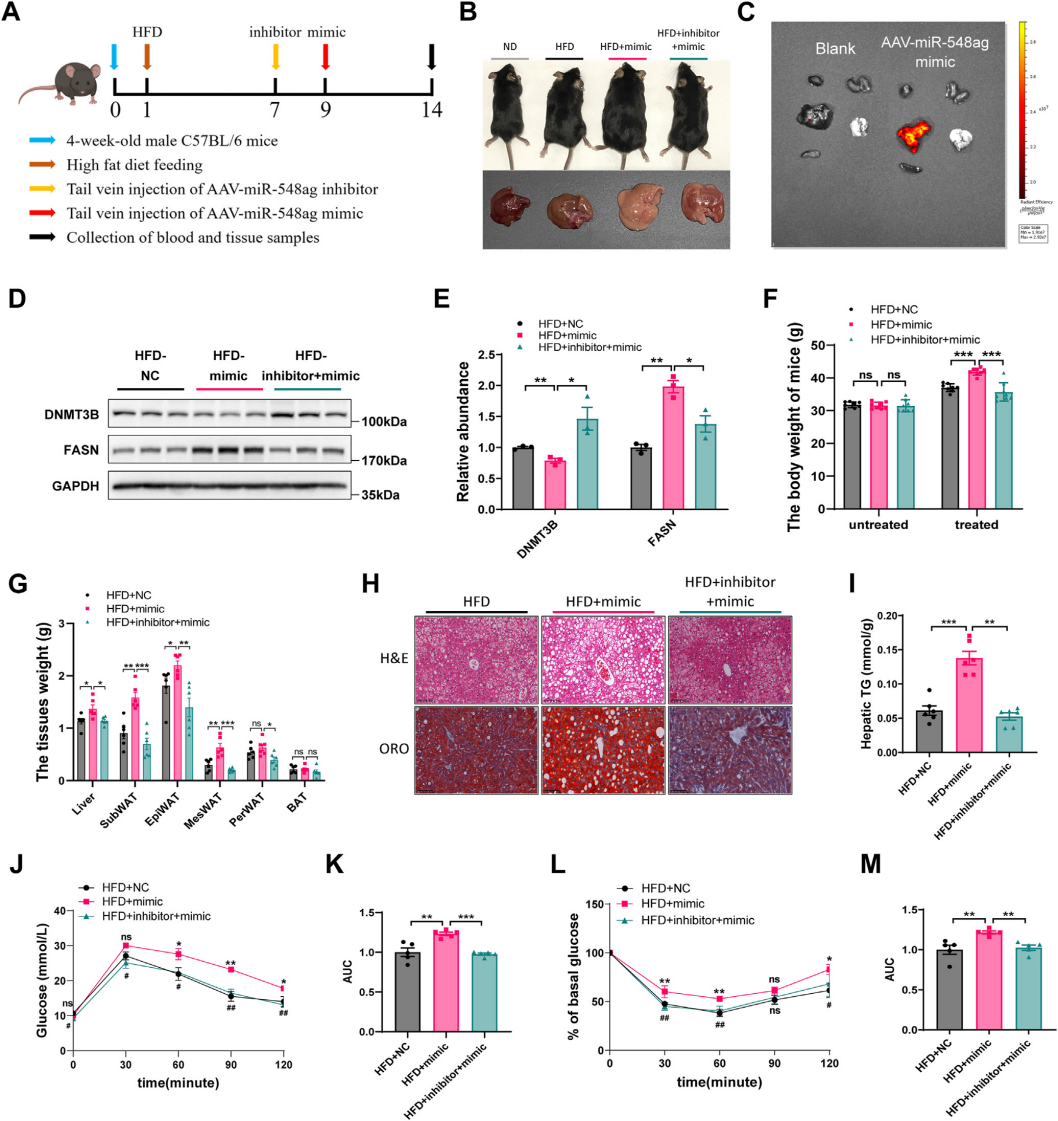
Effect of miR-548ag on protein and lipid accumulation in mouse liver. A: Time patterns of feeding and Adeno-associated virus treatment in C57BL/6 mice (n = 18). B: The gross morphology and liver of mice. C: IVIS imaging localization in various tissues of mice after tail vein injection of *mCherry*-labeled adeno-associated virus. D, E: Protein expression levels of DNMT3B and FASN in mouse liver. F: Comparison of body weights of mice before and after tail vein injection of adeno-associated virus (n = 6). G: Mouse liver and adipose tissue weights (n = 6). H: Oil red O and HE staining of mouse liver. I: TG content of mouse liver. J, K: Mouse GTT and area under the curve (n = 6). L, M: Mouse ITT and area under the curve (n = 6) (The *P*-values by the *t* test and nonparametric rank sum test are indicated. Data presented as mean ± sem. ∗*P* < 0.05, ∗∗*P* < 0.01, and ∗∗∗*P* < 0.001 indicate a significant difference, figures J and L, HFD+mimic group versus HFD+NC group, ∗*P* < 0.05, ∗∗*P* < 0.01, and ∗∗∗*P* < 0.001, HFD+inhibitor+mimic group versus HFD+mimic group, #*P* < 0.05, ##*P* < 0.01, ###*P* < 0.001, indicate a significant difference).

To investigate the in vivo role of miR-548ag in obesity, we fed C57BL/6 mice a HFD for 9 weeks, followed by tail-vein injection of *mCherry*-labeled *miR-548ag* mimic adenovirus serotype 8 (AAV8). The mice continued on the HFD until week 14 (Fig. 4A, B). In vivo imaging revealed that the miR-548ag mimic was predominantly enriched in the liver tissue of the treated mice (Fig. 4C). Following miR-548ag overexpression, we observed a significant decrease in the protein levels of DNMT3B and a marked increase in FASN expression in liver tissue (Fig. 4D, E). miR-548ag treatment also led to a significant increase in body weight and fat mass. Consistent with these changes, liver weight was markedly elevated, and Oil Red O staining revealed increased lipid accumulation in the liver. Furthermore, hepatic TG levels were significantly higher in the miR-548ag–overexpressing mice (Fig. 4F–I).

Additionally, miR-548ag overexpression in obese mice impaired glucose tolerance and insulin sensitivity (Fig. 4J–M). However, when miR-548ag mimic was co-administered with its inhibitor, the effects of miR-548ag on lipid accumulation and metabolic dysfunction were significantly reversed (Fig. 4D–M).

These results suggest that miR-548ag plays a pivotal role in promoting hepatic lipid accumulation and disrupting glucose homeostasis in obesity. Importantly, our findings also indicate that targeting miR-548ag may offer a potential therapeutic strategy for managing obesity-related metabolic dysfunction.

### Exosome-mediated transfer of miR-548ag to the liver promotes lipid accumulation

**Fig. 5:**
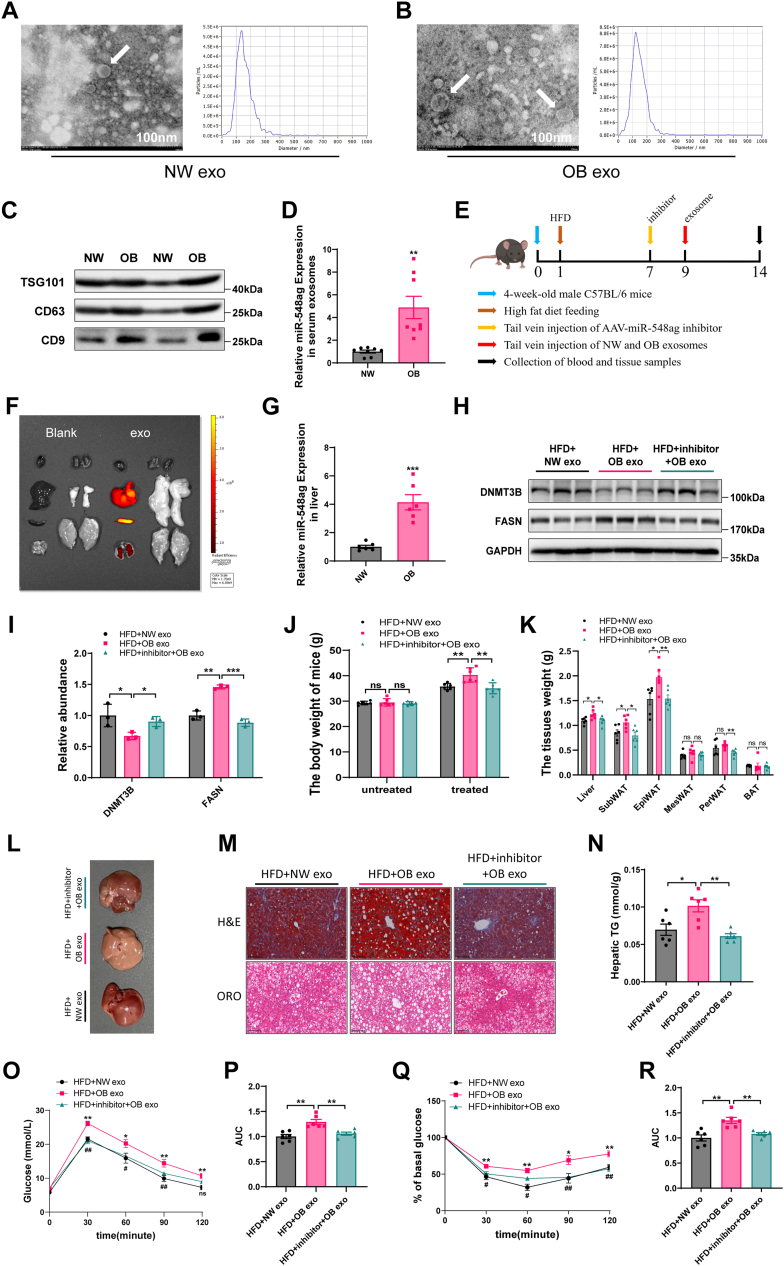
Effect of exosome miR-548ag on protein and lipid accumulation in mouse liver. A, B: Representative electron micrographs of exosomes extracted from serum and nanoparticle tracking analysis to determine exosome size distribution. C: Expression of extracted exosomal markers in the serum of equal numbers of individuals from the NW and OB groups was analyzed using Western blot methods. D: Comparison of miR-548ag content in serum exosomes of subjects in NW and OB groups. E: Temporal pattern of exosomal/adeno-associated virus treatment in C57BL/6 mice fed and injected in the tail vein (n = 18). F: IVIS imaging localization of DID-labeled exosomes in various tissues of mice after tail vein injection. G: Expression of miR-548ag in mouse liver (n = 6). H, I: Protein expression levels of DNMT3B and FASN in mouse liver. J: Comparison of body weights of mice before and after tail vein injection of exosomal/adeno-associated viruses (n = 6). K: Mouse liver and adipose tissue weights (n = 6). L: Pictures of mouse livers. M: Oil red O and HE staining of mouse liver. N: TG content of mouse liver. O, P: Mouse GTT and area under the curve (n = 6). Q, R: Mouse ITT and area under the curve (n = 6) (The *P*-values by the *t* test and nonparametric rank sum test are indicated. Data presented as mean ± sem. ∗*P* < 0.05, ∗∗*P* < 0.01, and ∗∗∗*P* < 0.001 indicate a significant difference, figures O and Q, HFD+OB exo group versus HFD+NW exo group, ∗*P* < 0.05, ∗∗*P* < 0.01, and ∗∗∗*P* < 0.001, HFD+inhibitor+OB exo group versus HFD+NW exo group, #*P* < 0.05, ##*P* < 0.01, ###*P* < 0.001, indicate a significant difference).

To determine whether miR-548ag is transported to the liver via exosomes and contributes to lipid accumulation through the upregulation of FASN, we isolated serum exosomes from normal-weight and obese individuals using ultracentrifugation. Transmission electron microscopy and nanoparticle tracking analysis confirmed successful exosome isolation, with particles exhibiting an average size of 100 nm (Fig. 5A, B). Western blot analysis of exosomal markers revealed that the expression of TSG101, CD63, and CD9 was significantly higher in obese individuals than normal-weight controls (Fig. 5C). Moreover, the miR-548ag content in serum exosomes was markedly elevated in obese individuals (Fig. 5D).

To investigate the functional impact of these exosomes, C57BL/6 mice were fed a HFD for 9 weeks and then injected via tail vein with serum exosomes derived from either NW (exo) or OB (exo) individuals. A third group received exosomes from obese individuals along with miR-548ag inhibitor treatment (inhibitor + OB, exo) (Fig. 5E). Small animal fluorescence imaging revealed that the injected exosomes predominantly accumulated in the liver (Fig. 5F). Notably, mice injected with OB-derived exosomes exhibited significantly higher hepatic miR-548ag levels than the NW exo group (Fig. 5G).

This elevation in miR-548ag corresponded to a significant decrease in DNMT3B expression and an increase in FASN protein levels in liver tissue (Fig. 5H, I). Additionally, OB, exo-treated mice showed increased body weight, liver weight, and adipose tissue mass, along with pronounced hepatic lipid accumulation as evidenced by Oil Red O staining and elevated TG levels (Fig. 5J–N). GTT and ITT further demonstrated that OB, exo treatment impaired glucose tolerance and insulin sensitivity (Fig. 5O–R).

Importantly, co-administration of miR-548ag inhibitor with OB-derived exosomes significantly reversed these phenotypes. miR-548ag inhibition restored DNMT3B expression, reduced FASN levels, mitigated hepatic lipid accumulation, and improved glucose tolerance and insulin sensitivity in treated mice (Fig. 5H–R).

### The impact of miR-548ag and DNMT3B on the methylation of CpG sites in the *FASN* promoter

**Fig. 6:**
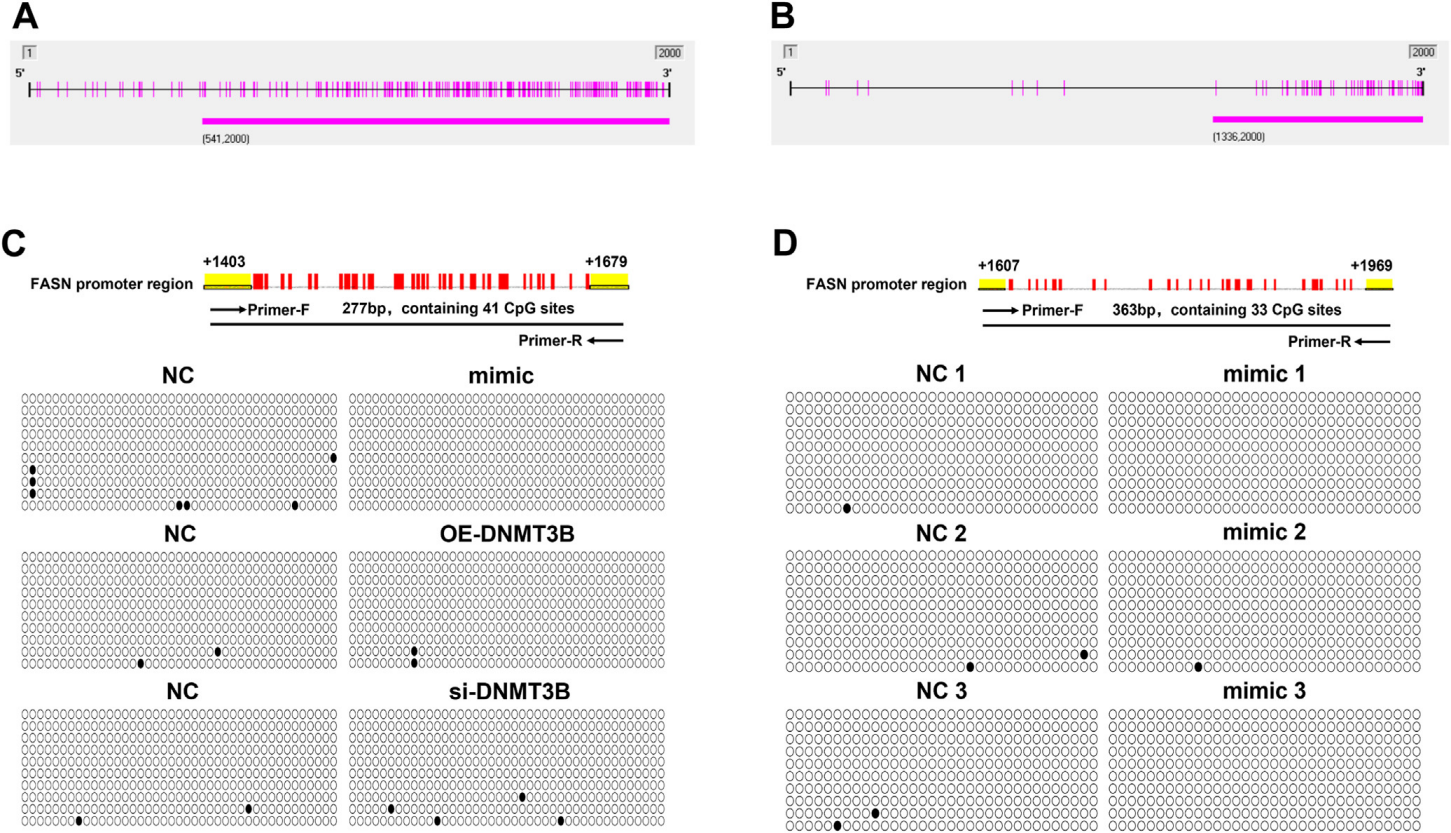
BSP experiment. A, B: CpG islands in the promoter region of the human and murine *FASN* genes were analyzed using metal prime express_V1 software. C: Schematic representation of the methylation status of the CpG site in the promoter region of the *FASN* gene after upregulation of miR-548ag and up/downregulation of DNMT3B in HepG2 cells. D: Schematic representation of the methylation status of the CpG site in the promoter region of the mouse liver *Fasn* gene after tail vein injection of miR-548ag overexpressing adeno-associated virus. (○ represents unmethylated CpG sites, • represents the CpG site where methylation occurs.)

Previous studies have demonstrated that miR-548ag directly targets DNMT3B and suppresses its expression (32). Based on bioinformatic analysis conducted using Methyl Primer Express v1, we identified CpG islands in the *FASN* promoter regions of both human and mouse genomes. These CpG islands exceeded 200 bp in length and had a GC content greater than 40% (Fig. 6A, B), suggesting a potential role for DNMT3B in regulating FASN expression via promoter methylation.

As a member of the DNA methyltransferase family, DNMT3B is primarily responsible for de novo DNA methylation. To explore whether FASN expression is regulated by methylation, we performed bisulfite sequencing experiments. Interestingly, the results revealed that overexpression of miR-548ag did not significantly alter the methylation status of CpG sites in the *FASN* promoter region in HepG2 cells (0% vs. 1.2%) (Fig. 6C and Supplemental Table S4).

Similarly, neither DNMT3B overexpression nor DNMT3B knockdown resulted in significant changes in the methylation of *FASN* promoter CpG sites in HepG2 cells (3.5% vs. 0% and 0% vs. 1.8%, respectively) (Fig. 6C and Supplemental Table S4). Moreover, consistent results were observed in miR-548ag–overexpressing mouse liver tissues, where the methylation status of CpG sites in the *Fasn* promoter region remained unchanged (0.5% vs. 0.5%) (Fig. 6D and Supplemental Table S4). These findings collectively suggest that the regulation of FASN expression by miR-548ag and DNMT3B does not occur through direct modulation of promoter methylation.

## Discussion

Previous studies have revealed a significant association between several members of the miR-548 family and tumorigenesis (36, 37, 38). However, research into the relationship between miR-548 family members and obesity or type 2 diabetes mellitus remains limited. Zheng Yang et al. reported a potential link between miR-548 family members and inflammation induced by obesity (39). Similarly, Samrita Mondal *et al.* found that miR-548 might influence appetite and leptin signaling (40). In a previous study conducted by our research group, single nucleotide polymorphism chip screening of 1,053 subjects revealed a significant positive correlation between miR-548ag and FPG levels. However, the biological function of miR-548ag in vivo remained unexplored. We subsequently discovered that miR-548ag is upregulated in obese individuals and contributes to glucose metabolism disorders by targeting DNMT3B, thereby upregulating DPP4 expression in the liver. Furthermore, we made a striking observation: administration of an adenovirus-packaged miR-548ag mimic to C57BL/6 male mice resulted in a significant increase in body weight and adipose tissue mass. Additionally, serum levels of TC, TG, FFA, and LDL were markedly elevated (32). These results strongly suggest that miR-548ag is not only involved in glucose metabolism but also plays a critical role in lipid metabolism dysregulation, highlighting its broader impact on metabolic homeostasis. This study uncovers a novel regulatory axis involving miR-548ag, DNMT3B, FASN, and hepatic lipid metabolism, a mechanism that has not been previously explored. These findings provide a new perspective on the broader metabolic functions of miR-548ag, extending beyond its known role in glucose homeostasis. By bridging the gap between glucose and lipid metabolism, this work highlights the multifaceted role of miR-548ag in metabolic regulation and underscores its potential as a key regulator in systemic metabolic disorders.

In the present study, we observed a significant elevation of miR-548ag levels in serum, adipose tissues, and liver tissues of obese and MASLD subjects. Serum miR-548ag levels were positively correlated with lipid parameters and liver function indicators, such as ALT and AST. Similarly, in HFD-fed mice, miR-548ag levels were markedly increased in serum, liver, and epididymal adipose tissues. Tail vein injection of miR-548ag mimic into HFD-fed mice resulted in pronounced lipid accumulation in the liver. Moreover, in vitro studies using human liver cancer cell line HepG2 and normal liver cell line L02 confirmed that miR-548ag upregulation promotes lipid synthesis. These findings suggest that obesity-induced miR-548ag elevation may be associated with lipid metabolism disorders and the development of MASLD. Elucidating the molecular mechanisms by which miR-548ag disrupts lipid metabolism could provide valuable insights into potential therapeutic targets for MASLD.

miRNAs are short noncoding RNAs that primarily regulate protein expression at the post-transcriptional level by targeting the 3′UTRs of mRNAs. A single miRNA can simultaneously regulate a wide range of genes due to its simple sequence-based targeting mechanism (41, 42, 43). Through bioinformatics analysis combined with RNA sequencing, we identified *FASN*, a key gene involved in lipid metabolism, as a potential downstream target of miR-548ag. Experimental evidence from liver tissue samples of human subjects, HFD-fed mice, and in vitro hepatocyte models confirmed that miR-548ag promotes lipid accumulation in the liver by upregulating FASN expression. Previous studies have shown that miRNAs typically bind to the 3′UTRs of target genes, leading to translational repression or mRNA degradation (44). However, in this study, miR-548ag unexpectedly enhanced the expression of its downstream target FASN, suggesting a noncanonical regulatory mechanism. Further investigation is warranted to decipher the precise molecular pathways underlying this phenomenon, which may unveil novel therapeutic strategies for lipid metabolism disorders.

DNA methylation, the earliest discovered and most fundamental epigenetic mechanism, involves genome-wide DNA hypomethylation and localized CpG island hypermethylation. It can alter chromatin structure, DNA conformation, stability, and interactions with proteins, ultimately influencing gene expression (45). Recent studies have highlighted that dysregulated lipid metabolism during obesity induces changes in the DNA methylation status of promoter regions in metabolic genes within adipose tissues, thereby affecting their expression (46, 47). A study by A. Young Kim *et al.* demonstrated that the inflammatory cytokine TNF-α significantly upregulates DNMT1 mRNA expression and enzymatic activity in cultured 3T3L1 adipocytes, leading to methylation of the *APN* gene promoter and subsequent repression of its expression (48). As the liver plays a central role in lipid metabolism, DNMT3B, a DNA methyltransferase highly expressed in liver tissue, has been implicated in epigenetic regulation. Recent research indicates that DNMT3B activity in HepG2 cells is modulated by miRNAs, subsequently affecting the methylation of downstream genes (49, 50). Bioinformatic analyses in this study identified DNMT3B as a potential target gene of miR-548ag, and experimental evidence demonstrated that DNMT3B knockdown significantly upregulates FASN expression. Sequence analysis of the *FASN* promoter revealed a high density of CpG sites (>40%). Further in vitro and in vivo experiments confirmed that miR-548ag suppresses DNMT3B expression, which in turn promotes FASN expression, leading to a marked increase in hepatic lipid synthesis. These results suggest that miR-548ag enhances *FASN* expression, potentially by modulating DNA methylation at its promoter. However, bisulfite sequencing (BSP) experiments revealed no significant changes in *FASN* promoter methylation when miR-548ag or DNMT3B were overexpressed. This finding indicates that the miR-548ag–mediated upregulation of *FASN* is not achieved through direct modification of its promoter’s methylation status. Thus, the observed regulatory effect of miR-548ag on FASN likely involves alternative mechanisms that bypass direct epigenetic modifications at the promoter. Identifying these mechanisms warrants further investigation to provide deeper insights into the interplay between miR-548ag, DNMT3B, and FASN in lipid metabolism regulation.

In this study, we utilized the PROMO database to predict potential transcription factors for *FASN* and identified 17 potential transcription factors. Using methylation primer design software (Methyl Primer Express_v1), we analyzed the promoter regions of these factors and discovered that 11 out of the 17 transcription factors, including *E2F1*, *SP1*, *IRF2*, *FOXA1*, *YY1*, *CEBPB*, *TBP*, *PAX5*, *MYC*, *NF1*, and *GCF*, contained CpG islands (Supplemental Fig. S3). Upon reviewing the literature, we found that the expression of all these genes, except *IRF2* and *GCF*, is regulated by DNA methylation (51, 52, 53, 54, 55, 56, 57, 58, 59, 60). Whether DNMT3B affects the expression of *FASN* through these transcription factors remains to be further explored.

MASLD is the most common cause of chronic liver disease (61). However, to date, no specific medications have been approved by the FDA or EMA for the treatment of MASLD (62, 63). Statins are recommended to improve cardiovascular outcomes and control hyperlipidemia in MASLD patients (64, 65). However, statin-induced liver toxicity remains a major safety concern, and regular liver function monitoring is required in chronic liver disease patients to avoid potential liver damage (66, 67, 68). Therefore, finding therapeutic approaches to treat or alleviate MASLD is of great significance. FASN is a key enzyme in hepatic de novo lipogenesis and is often upregulated in diseases such as nonalcoholic fatty liver disease and type 2 diabetes. The beneficial effects of hepatic FASN deficiency on nonalcoholic fatty liver disease and glucose metabolism are associated with the inhibition of de novo lipogenesis and the attenuation of gluconeogenesis and fatty acid oxidation, respectively (69, 70). Therefore, even minor changes in its activity or expression can severely disrupt the balance between lipid synthesis and degradation, leading to hepatic lipid accumulation (e.g., steatosis) or abnormal lipid distribution. Additionally, slight alterations in FASN may affect the lipid composition of hepatocyte membranes, as well as the function of the endoplasmic reticulum and mitochondria, thereby altering cell morphology and liver tissue structure (71, 72). Moreover, FASN dysfunction may trigger low-grade inflammation, which over time could contribute to liver fibrosis or cirrhosis, significantly changing the liver's appearance (73, 74). By modulating the expression of genes related to lipid metabolism, FASN may amplify the impact of its minor changes, resulting in substantial alterations in liver phenotype. Previous studies have shown that TRIM56 prevents MASLD by promoting FASN degradation (75), and SNX8 improves MASLD by enhancing FASN protein degradation (76). Additionally, research by Yang S *et al.* found that TAF15 directly interacts with the *FASN* promoter to enhance its expression, thereby promoting the development of MASLD (77). In this study, we constructed a miR-548ag overexpression mouse model and a mouse model injected with exosomes from the serum of obese individuals. We observed that overexpression of miR-548ag or injection of obese individual serum exosomes significantly increased mouse body weight and hepatic lipid accumulation. These changes were significantly alleviated upon administration of miR-548ag inhibitors. These results suggest that miR-548ag may serve as a potential therapeutic target for obesity and MASLD.

In summary, this study confirmed, both in HepG2 and L02 cell cultures and in a diet-induced obese mouse model, that miR-548ag can promote liver lipid accumulation by targeting and downregulating DNMT3B expression, which in turn upregulates FASN expression. Moreover, we established that the effect of DNMT3B on *FASN* expression is not mediated by direct changes in the methylation status of the CpG sites within its promoter region. Finally, we discovered that miR-548ag inhibitors could alleviate the hepatic lipid accumulation induced by a HFD in mice. Based on these findings, further evaluation of miR-548ag as a diagnostic and therapeutic target for MASLD is warranted, and this study provides experimental data and a theoretical foundation for the screening of new drug targets.

## Data Availability

All data are included in the article and supplemental information, and all data used in the article are available from the corresponding author upon reasonable request.

## Supplemental data

This article contains supplemental data.

## Conflicts of interest

The authors declare that they have no conflicts of interests with the contents of this article.
