# Supplementary material for: Exosome-derived miR-548ag drives hepatic lipid accumulation via upregulating FASN through inhibition of DNMT3B
Source: J Lipid Res. 2025 May 6;66(6):100818. doi: 10.1016/j.jlr.2025.100818 (PMC12164036; doi:10.1016/j.jlr.2025.100818)
Supplement: Supplementary Figure 1-3 [file mmc1.docx]

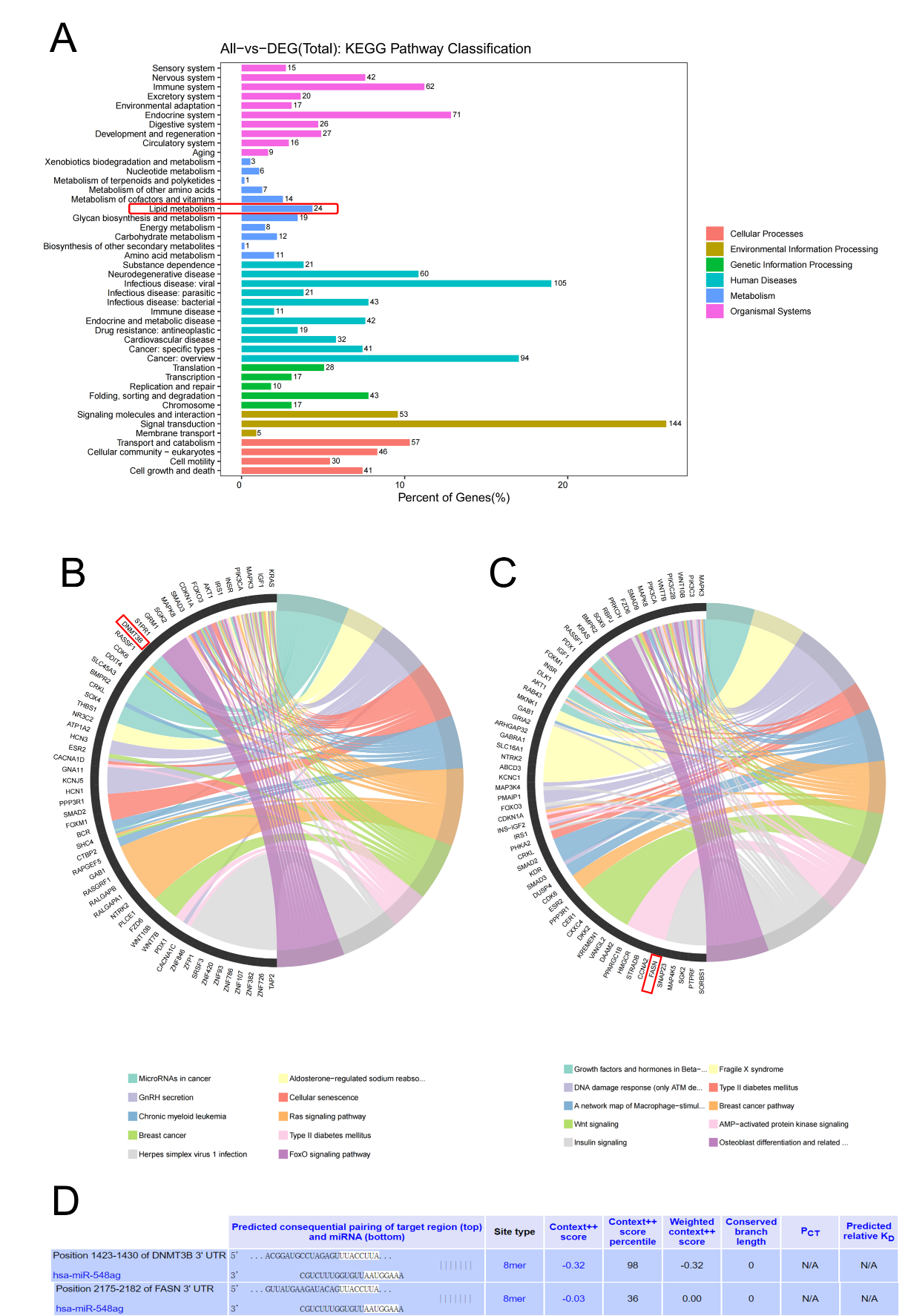


**Supplementary Fig. S1**

**Supplemental Fig. S1** Screening of miR-548ag target genes.

A-D: Analysis of miR-548ag enrichment pathway and screening of target genes using TatgetScan database combined with KEGG enrichment analysis.


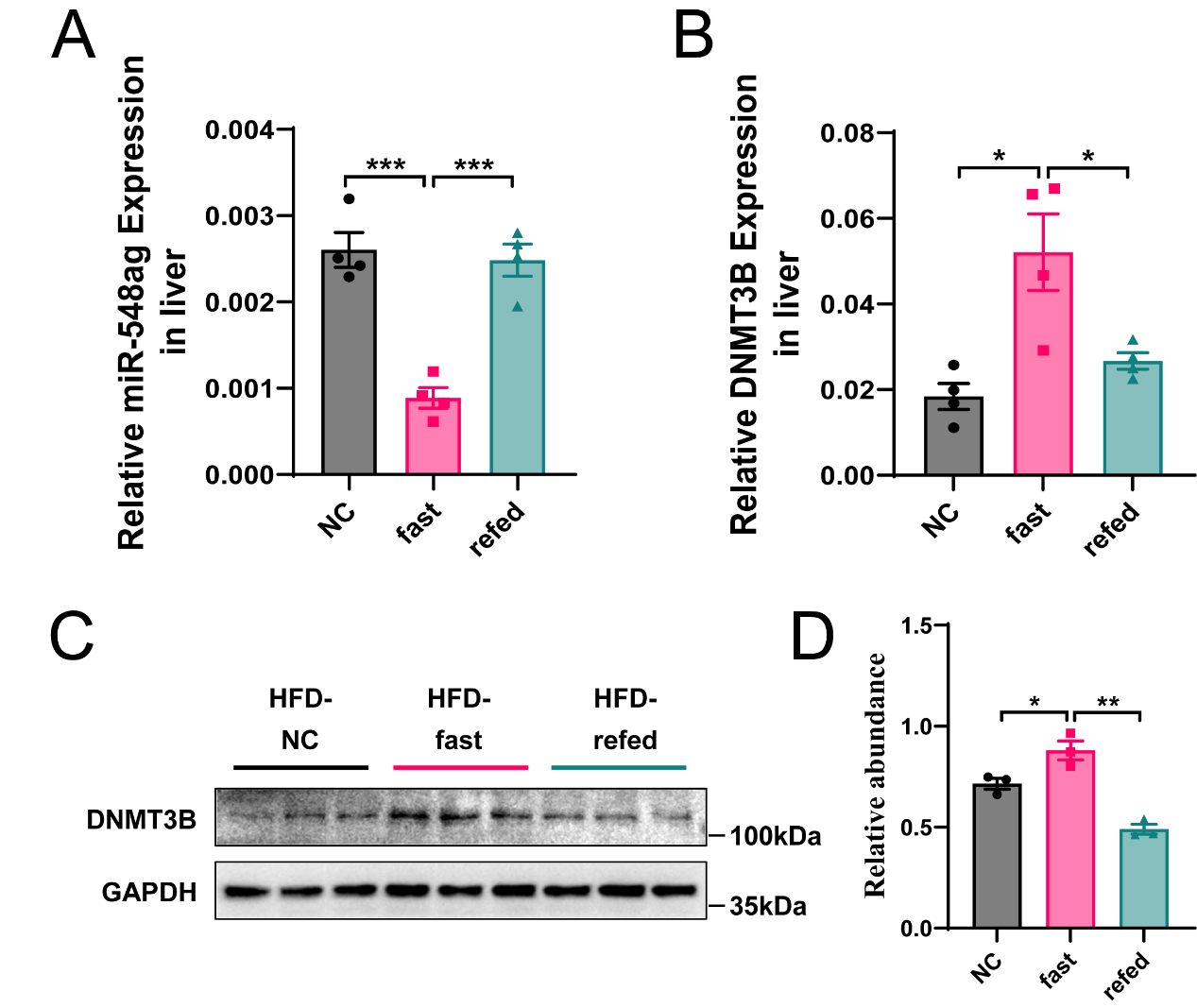


**Supplementary Fig. S2**

**Supplemental Fig. S2.** The effects of fasting and refeeding on miR-548ag and DNMT3B expression levels.

A: Expression of miR-548ag in mouse liver. B: Expression of DNMT3B mRNA in mouse liver. C-D: Protein expression and quantification results of DNMT3B in mouse liver. (The *p*-values by the *t*-test and non-parametric rank sum test are indicated. Data presented as mean ± sem. **P*<0.05, ***P*<0.01, and ****P*<0.001 indicate a significant difference)


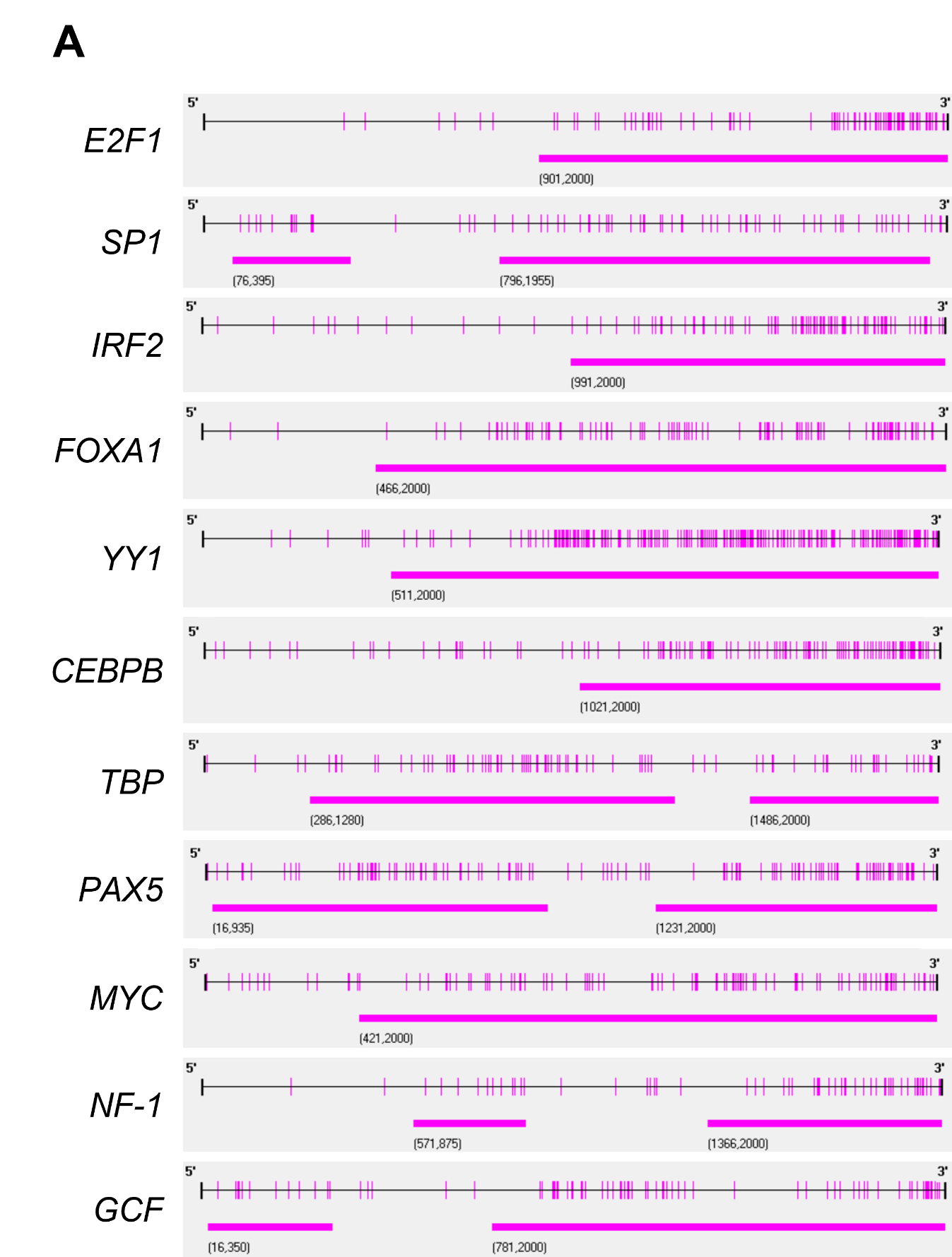


**Supplementary Fig. S3**

**Supplemental Fig. S3.** *FASN* Transcription Factor GpG Island. A Use metal prime express_ V1 software analyzes the CpG island of *E2F1*, *SP1*, *IRF2*, *FOXA1*, *YY1*, *CEBPB*, *TBP*, *PAX5*, *MYC*, *NF1* and *GCF*.
