# Supplementary material for: Exosome-derived miR-548ag drives hepatic lipid accumulation via upregulating FASN through inhibition of DNMT3B
Source: J Lipid Res. 2025 May 6;66(6):100818. doi: 10.1016/j.jlr.2025.100818 (PMC12164036; doi:10.1016/j.jlr.2025.100818)
Supplement: Supplementary Table 1-4 [file mmc2.docx]

**Supplementary Table S1 Primer sequence table**

| Species | primer | sequence 5’→3’ |
| --- | --- | --- |
| Human | Homo-*microRNA-548ag*-F | AAAGGUAAUUGUGGUUUCUGC |
|  | Homo-*DNMT3B*-F | GGATGAAGATCAGAGCCGA |
|  | Homo-*DNMT3B*-R | CAAGACAAACAGCCATCTTCC |
|  | Homo-*FASN*-F | ACAGCGGGGAATGGGTACT |
|  | Homo-*FASN*-R | GACTGGTACAACGAGCGGAT |
|  | Homo-*GAPDH*-F | TGTGGGCATCAATGGATTTGG |
|  | Homo-*GAPDH*-R | ACACCATGTATTCCGGGTCAAT |
|  | Homo-*U6*-F | CTCGCTTCGGCAGCACAT |
|  | Homo-*U6*-R | TTTGCGTGTCATCCTTGCG |
| Mouse | Mus-*DNMT3B*-F | CGCACAACCAATGACTCTGCTG |
|  | Mus-*DNMT3B*-R | GGTGACTTCAGAAGCCATCCGT |
|  | Mus-*FASN*-F | CACAGTGCTCAAAGGACATGCC |
|  | Mus-*FASN*-R | CACCAGGTGTAGTGCCTTCCTC |
|  | Mus-*GAPDH*-F | CATCACTGCCACCCAGAAGACTG |
|  | Mus-*GAPDH*-R | ATGCCAGTGAGCTTCCCGTTCAG |
|  | Mus-*U6*-F | CGCTTCGGCAGCACATATAC |
|  | Mus-*U6*-R | AAATATGGAACGCTTCACGA |
| other | Cel- *microRNA-39*-F | UCACCGGGU GUAAAUCAGCUUG |

**Supplementary Table S2 Correlation analysis between miR-548ag**

**and Serum biochemicalparameters of individuals**

| Index  (n=52) | miR-548ag | |
| --- | --- | --- |
|  | R | P-value |
| BMI | 0.442 | 0.001 |
| FPG | 0.436 | 0.001 |
| TC | 0.361 | 0.009 |
| TG | 0.380 | 0.005 |
| LDL | 0.384 | 0.005 |
| HDL | -0.013 | 0.929 |

Pearson correlation analysis, *P*<0.01, the correlation has statistical significance.

**Supplementary Table S3 General information of subjects**

|  | NW  (n=41) | OB  (n=18) | MASLD  (n=24) |
| --- | --- | --- | --- |
| Age (year) | 46.93±13.73 | 56.17±12.63* | 49.71±12.70 |
| Height (cm) | 162.95±9.34 | 162.39±7.55 | 165.10±8.08 |
| Weight (kg) | 58.01±8.40 | 88.85±23.49*** | 89.05±9.05*** |
| BMI(kg/m^2^) | 21.8±2.06 | 33.43±6.98*** | 32.67±2.50*** |
| Waistline (cm) | 75.70±8.26 | 103.31±21.84*** | 105.33±15.88*** |
| FPG (mmol/L) | 5.55±1.35 | 5.88±1.15 | 5.80±0.86 |
| TC (mmol/L) | 4.32±0.97 | 5.13±0.90** | 4.71±0.60** |
| TG (mmol/L) | 1.07±0.45 | 1.61±0.66** | 1.86±1.33* |
| LDL (mmol/L) | 2.38±0.91 | 3.35±1.08*** | 2.99±0.83** |
| HDL (mmol/L) | 1.49±0.36 | 1.40±0.29 | 1.33±0.23 |

T-test, rank sum test, compared with NW group, **P*<0.05, ***P*<0.01, ****P*<0.001, the difference was statistically significant.

**Supplementary Table S4 Effects of miR-548ag and DNMT3B on methylation of CpG site in promoter region of *FASN* gene in HepG2 cells and liver**

|  | Group | Methylation rate | Demethylation rate |
| --- | --- | --- | --- |
| HepG2 | miR-548ag-NC | 1.7% (7/410) | 98.3% (403/410) |
|  | miR-548ag-mimic | 0 | 100% (410/410) |
|  | OE-DNMT3B-NC | 0.5% (2/410) | 99.5% (408/410) |
|  | OE-DNMT3B | 0.5% (2/410) | 99.5% (408/410) |
|  | si-DNMT3B-NC | 0.5% (2/410) | 99.5% (408/410) |
|  | si-DNMT3B | 1.0% (4/410) | 99.0% (406/410) |
| Liver | miR-548ag-mimic-NC | 0.5% (1.7/330) | 99.5% (328.3/330) |
|  | miR-548ag-mimic | 0.1% (0.3/330) | 99.9% (329.7/330) |
|  | miR-548ag-inhibitor-NC | 1.1% (3.6/330) | 98.9% (326.4/330) |
|  | miR-548ag-inhibitor | 1.3% (4.3/330) | 98.7% (325.7/330) |

χ*^2^* test, *P*>0.05.
